# Knockdown of the Ribosomal Protein eL38 in HEK293 Cells Changes the Translational Efficiency of Specific Genes

**DOI:** 10.3390/ijms22094531

**Published:** 2021-04-26

**Authors:** Alexander V. Gopanenko, Alena V. Kolobova, Alexey E. Tupikin, Marsel R. Kabilov, Alexey A. Malygin, Galina G. Karpova

**Affiliations:** Institute of Chemical Biology and Fundamental Medicine, Siberian Branch of the Russian Academy of Sciences, Prospekt Lavrentieva 8, 630090 Novosibirsk, Russia; alexandr.gopanenko@yandex.ru (A.V.G.); alyona_kolobova@rambler.ru (A.V.K.); alenare@niboch.nsc.ru (A.E.T.); kabilov@niboch.nsc.ru (M.R.K.); malygin@niboch.nsc.ru (A.A.M.)

**Keywords:** HEK293 cells, knockdown of ribosomal protein eL38, next-generation sequencing, Ribo-seq, genes with eL38-dependent translational efficiencies, eL38-related processes

## Abstract

The protein eL38 is one of the smallest proteins of the mammalian ribosome, which is a component of its large (60S) subunit. The haploinsufficiency of eL38 in mice leads to the Tail-short mutant phenotype characterized by defects in the development of the axial skeleton caused by the poor translation of mRNA subsets of Hox genes. Using the ribosome profiling assay applied to HEK293 cells knocked down of eL38, we examined the effects of the lack of eL38 in 60S subunits on gene expression at the level of translation. A four-fold decrease in the cell content of eL38 was shown to result in significant changes in the translational efficiencies of 150 genes. Among the genes, whose expression at the level of translation was enhanced, there were mainly those associated with basic metabolic processes; namely, translation, protein folding, chromosome organization, splicing, and others. The set of genes with reduced translation efficiencies contained those that are mostly involved in the processes related to the regulation of transcription, including the activation of Hox genes. Thus, we demonstrated that eL38 insufficiency significantly affects the expression of certain genes at the translational level. Our findings facilitate understanding the possible causes of some anomalies in eL38-deficient animals.

## 1. Introduction

In all living organisms, protein synthesis (translation) is carried out by ribosomes, which are supramolecular ribonucleoprotein complexes that polymerize amino acids into proteins in accordance with the codon sequences in the translated mRNAs. The mammalian ribosome contains 80 different proteins [1,2], which, being integrated into its large (60S) and small (40S) subunits during the assembly and maturation of the latter, ultimately form their own specific structures that are involved to one degree or another in ensuring the operation of the translational machinery. Mutations in the genes of certain ribosomal proteins, leading to their cellular haploinsufficiency, cause aberrations in the assembly of ribosomal subunits and are associated with a number of serious diseases, collectively referred to as ribosomopathies [3]. Besides, there are several observations that in mammalian ribosomes the levels of some proteins are significantly reduced compared to those of all other ribosomal proteins [4,5]. This means that mammalian cells contain subpopulations of active ribosomes, heterogeneous in protein composition, which, according to the concept of specialized ribosomes [6] arising from the ribosome filter hypothesis [7], can preferentially translate different subsets of mRNAs.

The studies that have led to the above concept concern the ribosomal protein eL38. This protein is located on the surface of the 60S subunit in the region opposite the central protuberance, approximately at the same distance from the nascent peptide exit site and from the feet of the 40S subunit, far away from the key functional centers of the ribosome [2]. Phenotypic manifestations of eL38 haploinsufficiency, representing various skeletal abnormalities, including a shortened tail [8], 14 pairs of thoracic ribs instead of 13 ones, and some others [9], have been shown in Ts mutant mice with a spontaneous deletion of the 18 kb fragment covering the *Rpl38* locus. It has been suggested that eL38 is engaged in the establishment of the mammalian body plan during embryogenesis by selectively facilitating the translation of specific mRNA subsets of Hox genes that regulate the axial skeleton morphology, which turned out to be suppressed in these mice [9,10]. Besides, the expression of *Rpl38* has been found to exhibit a tissue-specific pattern and to be highly variable in different cells during organogenesis [9]. All this implies that eL38 is a player in the processes related to the regulation of gene expression, and that its deficiency can lead to their reorganization through changes in the translational activities of certain mRNA sets in accordance with the concept of specialized ribosomes, which should facilitate the adaptation of cells to these conditions. The existence of this kind of regulation is indicated by the data of quantitative proteomic analysis performed on mouse embryonic stem cells showing that the level of ribosomal protein eL38 in the polysome fraction collected after the sedimentation of the cell lysate in a sucrose density gradient was approximately 0.6 of the levels of most other proteins of the 60S ribosomal subunit [5].

In our previous work using the RNA-seq approach, we have shown that the knockdown of eL38 in HEK293 cells caused a substantial reorganization of genomic transcription, resulting in the altered expression of nearly 1500 genes [11]. An analogous effect of eL38 deficiency on gene expression has been demonstrated in HepG2 cells using RT-qPCR for a group of genes identical to those whose transcriptional activities were reduced or enhanced in HEK293 cells with the decreased level of eL38, which suggests that the response of different cell types to eL38 insufficiency is generally similar [11]. In this study, using the ribosome profiling method (Ribo-seq), we investigated how the reduced content of eL38 in HEK293 cells affects gene expression at the level of translation. This method is based on the isolation of fragments of mRNAs translated at a given moment, which are protected from RNase hydrolysis by ribosomes, and on their subsequent high throughput sequencing [12], which makes it possible to determine the translational efficiencies (TEs) of genes regardless of contents of their mRNAs in cells. Using Ribo-seq, we revealed genes with increased and decreased TEs, which allowed for the identification of particular cellular processes, whose regulation is associated with these genes. Our results led us to the conclusion that the cell’s strategy to adapt to eL38 deficiency is to enhance the expression at the translational level of a certain and rather limited set of multifunctional genes that activate cellular metabolism. Along with this, there is also a suppression of translational activities of some genes related to the regulation of transcription and, in particular, to the activation of Hox genes, which may be of importance for the processes of development and differentiation.

## 2. Results

### 2.1. Ribo-seq of eL38 Knocked down HEK293 Cells

**Figure 1:**
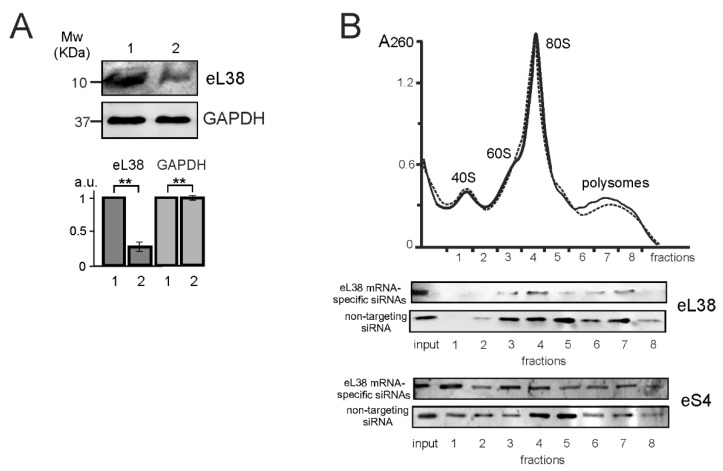
Ribosomal protein eL38 knockdown in HEK293 cells. (**A**) Western blot analysis of the levels of eL38 and GAPDH (as a reference) in cells transfected with siRNAs specific for eL38 mRNA (1) and non-targeting siRNA (2). The diagram shows triplicate western blot data as the mean of arbitrary units (a.u.) ± SEM (** *p* < 0.01, calculated using Mann-Whitney test). (**B**) Sucrose gradient polysome profiles obtained by the centrifugation of the lysates of cells transfected with non-targeting siRNA (solid line) and eL38 mRNA-specific siRNAs (dashed line); the peaks of 60S and 40S subunits, 80S ribosomes and polysomes are marked. Western blot analysis of the sucrose gradient fractions for the presence of ribosomal proteins eL38 and eS4 (as a reference).

The knockdown of eL38 in HEK293 cells was performed using an RNA interference assay. To this end, cells were transfected with specific siRNAs targeting the coding sequence (CDS) of eL38 mRNA, utilizing cells transfected with non-targeting siRNA as a control. After two days, the total level of eL38 in cells treated with specific siRNAs decreased by 4 times (Figure 1A). As shown in our previous work using the MTT test [11], the treatment of HEK293 cells with the above siRNAs practically did not reduce their viability. An analysis of the eL38 contents in the ribosome and polysome fractions after the centrifugation of total cell lysates in a sucrose density gradient showed a considerable decrease in the relative levels of eL38 in the fractions corresponding to cells transfected with specific siRNAs (Figure 1B). This implied that the eL38 knocked down cells contained a significant number of ribosomes lacking eL38, which were associated with actively translated mRNAs in polysomes, and therefore were suitable for studying the effects of reduced eL38 content on the translatome profile.

For the Ribo-seq procedure, the eL38 knocked down cells were obtained from HEK293 ones cultured in three biological replicates. The subsequent manipulations were cell lysis, the treatment of the lysate with RNase I to digest the polysomes into monosomes, followed by the collection of the latter by passing through a sucrose cushion using ultracentrifugation and the isolation of ribosome-protected mRNA fragments, from which DNA library was prepared for high throughput sequencing. Analysis of sequencing reads showed that they mainly fell into those genomic regions that correspond to the CDSs of mRNAs (Figure S1), and the distribution of their lengths was predominantly in the range of 28–32 nt, which is in accordance with the length of mRNA fragments protected by the ribosome from RNase I hydrolysis (Figure S2). In addition, the mapping of Ribo-seq reads revealed triplet periodicity in DNA sequences, which is a signature that ribosome-protected mRNA fragments originate from translated mRNAs (Figure S2). All this indicated that the Ribo-seq data quality was good enough for downstream analysis, which resulted in the data summarized in Table S1.

### 2.2. Identification of Genes with Differential Translational Efficiencies

**Figure 2:**
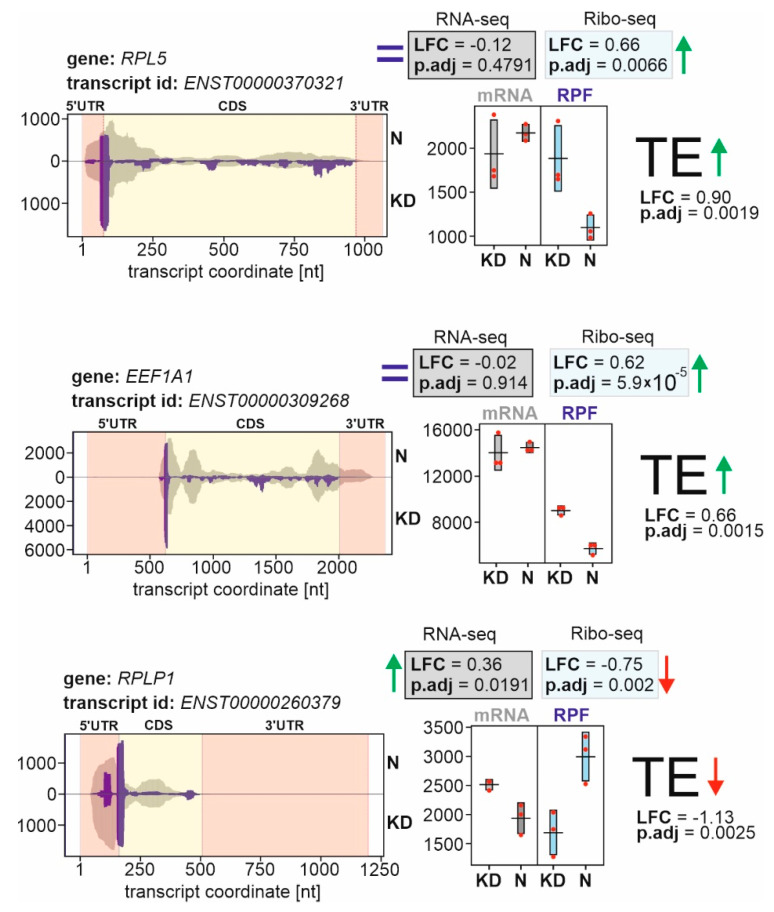
The coverage plots for three representative genes with differential translational efficiencies (GDTEs) in HEK293 cells treated with specific siRNAs against eL38 mRNA and with non-targeting siRNA. The left panels show the normalized read coverage of gene regions, given in coordinates of the respective transcripts, for cells with normal eL38 level (the upper part, N) and eL38 knocked down cells (the lower part, KD). The brown and purple histograms on the plots illustrate the read densities across these regions derived from the RNA-seq and Ribo-seq data, respectively. The yellow and peach areas on the plots correspond to the coding sequences (CDSs) of transcripts and their 5′- and 3′-untranslated regions (UTRs), respectively. The right panels present the dot plots of normalized read counts obtained from the RNA-seq (“mRNA” plot) and Ribo-seq (“RPF” plot) data in three replicates. The DESeq2-derived statistics, Log2FoldChange (LFC) and *p* adjusted (*p*.adj) values, are given inside the boxes. The TE change for each gene is designated by an arrow and its statistics are shown.

To determine the effects of eL38 deficiency on the translatome profile, the Ribo-seq was performed in combination with the high throughput sequencing of the total cellular mRNA (RNA-seq) [11]. The TE parameters of genes were calculated taking into account the Ribo-seq and RNA-seq data obtained for cell samples treated with specific siRNAs against eL38 mRNA and with non-targeting siRNA. This made it possible to identify genes with altered TEs, regardless of whether their expression increased or decreased at the transcriptional level and, accordingly, whether the total cellular mRNA level changed. To sort the genes with the most pronounced and statistically significant changes in their TEs from the obtained set, we limited the shrunken Log2 Fold Change (LFC) parameters to an absolute value of more than 0.585 (which corresponded to the changes of more than 50%), and the *p* adjusted (*p*.adj) values were no more than 0.1. In total, these criteria were met by 150 genes, whose expression at the translation level was enhanced or reduced (Table 1 and Table 2, respectively). Genes with differential TEs in cells knocked down of eL38 and in cells with normal eL38 level are hereinafter referred to as GDTEs. Data from the analysis of gene TE parameters are presented in Figure 2 using the example of three selected GDTEs.

### 2.3. Genes with Increased TEs and Associated Cellular Processes

Among the up-regulated GDTEs (Table 1), there were those related to basic cellular processes. In particular, these were genes *RPL26*, *RPL10*, *RPL5*, *RPS24*, *RPS15A* and *RPS3A* encoding ribosomal proteins, as well as *EIF4A2*, *EIF3L*, *EIF5*, *EIF4H*, *EIF2S3* and *EEF1A1* encoding translation factors. In addition, this GDTE set included *HSPA9* encoding a chaperone involved in stress-mediated protein folding, *CCT3*, *CCT4*, *CCT6A* and *CCT7* encoding components of the chaperonin-containing T-complex, as well as *LAMP2* encoding a protein implicated in chaperone-mediated autophagy. The genes, such as *RCC1* and *HMGB1* encoding proteins involved in the formation of chromatin, *HNRNPA3*, *HNRNPA1* and *HNRNPA2B1* encoding players in the processing and splicing of pre-mRNAs, as well as *SHMT2*, *ATP5MG* and *PMPCB* encoding mitochondrial enzymes and *CDK1* encoding a key protein kinase that controls the cell cycle, were also activated.

**Figure 3:**
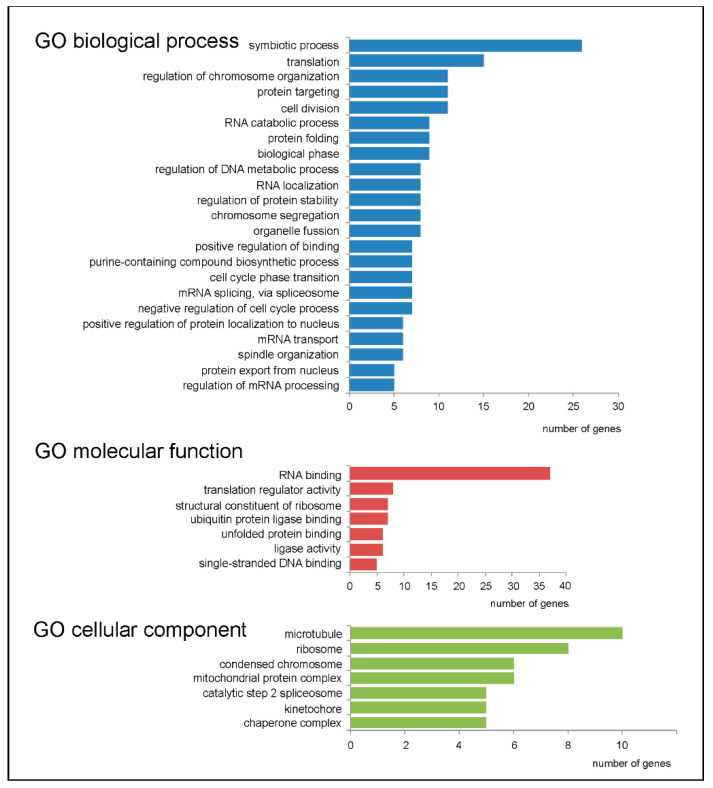
Gene ontology (GO) analysis performed for the up-regulated GDTEs set from HEK293 cells knocked down of eL38. Only the highest categories in the GO hierarchy (biological process, molecular function, and cellular component) with multiple enrichment values > 5 and gene numbers of at least 5 are presented.

**Figure 4:**
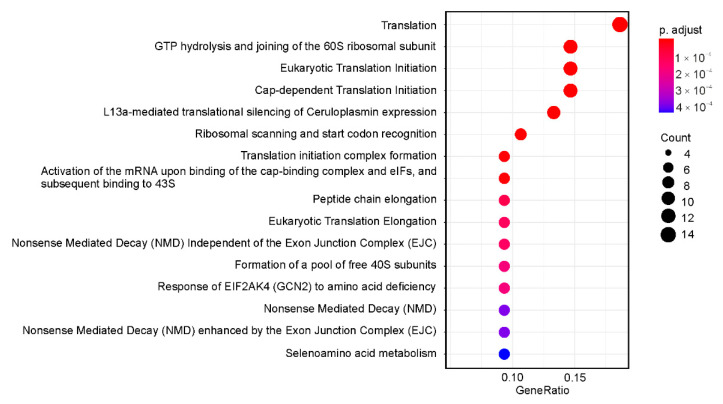
Dotplot enrichment map showing cellular pathways associated with up-regulated GDTEs from HEK293 cells knocked down of eL38. The colors of the points depend on the *p*.adj values, and their sizes are determined by the number of GDTEs associated with the corresponding pathways (color and dot size keys are shown on the right).

Gene ontology (GO) analysis, aimed at determining those biological processes, molecular functions and cellular components in which the up-regulated GDTEs are involved (Figure 3), revealed an enrichment for genes mainly associated with numerous primary metabolic processes. In particular, among GO biological processes were translation (*RARS*, *RPL5*, *EEF1A1* and others), the regulation of chromosome organization (*HNRNPA1*, *CCT4*, *KAT7* and others), protein folding (*HSPA9*, *CCT*, *B2M* and others) and symbiotic process (*CDK1*, *CADM1*, *HNRNPA2B1* and others). From GO molecular functions, there was an enrichment for the GDTEs encoding proteins attributing presumably to RNA binding (*NME1*, *SHMT2*, *RARS* and others), the activity of translation regulators (*EEF1A1*, *EIF5*, *EIF4H* and others) and the binding of unfolded proteins (*HSPA9*, *CCT4*, *CCT7* and others). Finally, for GO cellular components, an enrichment was found in GDTEs that are mostly related to microtubules (*TUBA1A*, *CCT4*, *CCT6A* and others), ribosomes (*RPL10*, *RPL5*, *RPS24* and others), mitochondrial protein complex (*ATP5MG*, *PMPCB*, *PDHA1* and others) and kinetochore (*NUP43*, *KIF22*, *SEPT7* and others). It should be noted that many up-regulated GDTEs were found to be enriched in multiple GO categories, which implies that their products can be engaged in several processes simultaneously. For example, *HNRNPA1* and *HNRNPA2B1* are implicated in the regulation of chromosome organization, symbiotic process, RNA localization, mRNA splicing via spliceosome, mRNA transport and several others, while *CCT3*, *CCT4* and *CCT7* are involved in protein folding processes, the regulation of protein stability, the positive regulation of protein localization in the nucleus and the regulation of the DNA metabolic process. Besides, *CDK1*, *SKP1* and *BUB1* are related to symbiotic process, biological phase and the negative regulation of the cell cycle process, whereas *NUP43* and *RCC1* are associated with cell division, symbiotic process, spindle organization and chromosome segregation. Reactome pathway-based analysis of the set of up-regulated GDTEs performed with the ReactomePA package also revealed a statistically significant enrichment for those associated with translation, nonsense mediated decay, response of eIF2AK4 (GCN2) to amino acid deficiency and related processes, and some others (Figure 4). Thus, a decrease in the level of the ribosomal protein eL38 in cells leads to an increase in the TEs of a relatively small number of genes, whose products are involved in the regulation of basic metabolic processes. The products of many of these genes can participate in several processes at once, which allows them to be considered to some extent as multifunctional metabolic regulators.

### 2.4. Genes with Decreased TEs and Associated Cellular Processes

The list of the down-regulated GDTEs from eL38 knocked down HEK293 cells (Table 2) was somewhat shorter than that for the up-regulated ones (Table 1). The calculation of the average CDS length for mRNAs of down-regulated GDTEs showed that it was about three times longer (~3800 nt) than that of mRNAs of protein-coding genes in the human genome (1278 nt) [13], indicating that the TEs of mRNAs with the long CDSs are especially decreased when ribosomes lacking eL38 appear in cells.

Among the down-regulated GDTEs were, for example, the collagen genes *COL2A1* and *COL6A1* implicated in the formation of cartilage tissue and in cell binding, respectively, *AUTS2* encoding a protein that activates transcription, controls development and is necessary to maintain the repressive state of transcription of many genes, as well as *CDK5RAP2* and *GAK*, which regulate the activity of CDK5 kinase, and other genes. The GO analysis of down-regulated GDTEs using the same search parameters as in the GO analysis of up-regulated GDTEs (Figure 3) revealed an enrichment for the genes *HMGA1*, *UPF1*, *GSK3A*, *KMT2D*, *HIST1H4E*, and *CTCF*, which turned out to be associated with only one biological process, namely, the epigenetic regulation of gene expression (number of genes is 6, fold enrichment is 11.23). However, no significant enrichment was found for down-regulated GDTEs in the categories of molecular function and cellular component.

**Figure 5:**
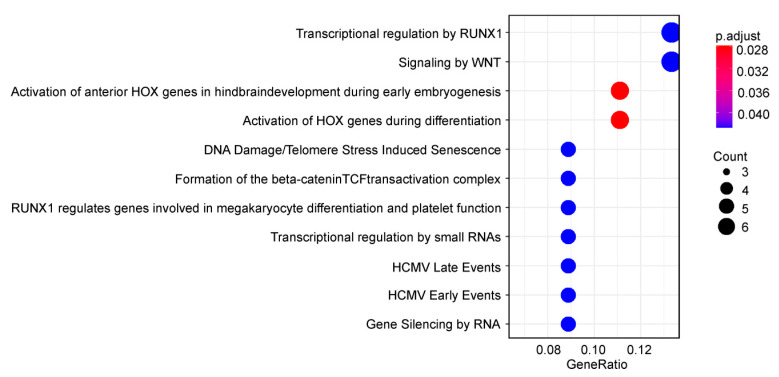
Dotplot enrichment map showing cellular pathways associated with down-regulated GDTEs from HEK293 cells knocked down of eL38. Designations are the same as in Figure 4.

Reactome pathway-based analysis of down-regulated GDTEs exhibited a statistically significant enrichment for those mainly associated with transcriptional regulation processes (Figure 5). Among them were signaling by WNT (*CLTC*, *HIST1H2AC*, *KMT2D*, *HIST1H4E*, *ITPR3* and *HIST1H2BC*), transcriptional regulation by RUNX1 (*HIST1H2AC*, *SMARCC1*, *KMT2D*, *HIST1H4E*, *HIST1H2BC* and *AUTS2*) and the activation of Hox genes during differentiation (*H2AC6*, *KMT2D*, *HIST1H4E*, *CTCF* and *HIST1H2BC*). The latter is especially noteworthy, because the effect of eL38 deficiency in ribosomes on the translation of subsets of Hox gene mRNAs has already been reported earlier [9]. Given the decrease in the TEs of the collagen genes *COL2A1* and *COL6A1* observed in our study, one can assume that eL38 insufficiency leads to a dysregulation of bone formation during development.

## 3. Discussion

The analysis of Ribo-seq data obtained on HEK293 cells, where the content of ribosomal protein eL38 was reduced by RNA interference, revealed significant changes in gene expression at the translational level compared to cells with normal eL38 content. We showed that an approximately 4-fold decrease in the level of eL38 in ribosomes did not considerably affect the overall polysome profile, but caused pronounced, statistically significant changes in TEs for one and a half hundred genes, indicating a modulation of translation on ribosomes lacking eL38. Activation was found to occur for 84 representative genes associated with the main cellular metabolic processes, such as translation, protein folding, mRNA processing and splicing, and others; many of these genes turned out to be implicated in several such processes. As for the set of 66 genes with reduced TEs, it consisted basically of those mainly associated with the processes of regulation of transcription, including the activation of Hox genes during differentiation, and the mRNA CDSs of the most of them were significantly longer than the average length of CDSs in the mRNAs of protein-coding genes in the human genome. The results obtained contribute to understanding how the mammalian cellular translatome can change upon eL38 deficiency and highlight the possible causes of impaired skeletogenesis at the early stages of development.

Although differentiating cells seem to be the most suitable models for investigating the causes of congenital anomalies associated with eL38 haploinsufficiency in mutant mice, the use of standard HEK293 cells allowed us to determine the general consequences of a deficiency of this protein on the cellular translatome. As mentioned in the Introduction, we have previously shown that a decrease in the eL38 level in HEK293 cells leads to a change in the expression (activation or suppression) of more than 1500 genes at the transcriptional level [11]. This means that the cellular response to eL38 deficiency is more pronounced at the transcriptional level, rather than at the translational one. Nevertheless, the processes that were revealed for genes whose expression was enhanced at the level of translation were largely similar to those that have been found to be associated with genes activated at the transcriptional level. These are, first of all, the processes related to translation and splicing. At the same time, among the translationally activated genes, there are no those involved in translation in mitochondria, which are present in the set of transcriptionally activated genes [11]. On the other hand, the expression of genes associated with protein folding was increased at the level of translation, while they have been shown not to be included in the number of transcriptionally activated genes. As for the genes whose TEs were suppressed, they were mainly implicated in the regulation of transcription, whereas those that have been found to become less active at the transcription level were more related to signaling and only partially to transcriptional regulation.

All of the above indicates that the deficiency of the ribosomal protein eL38 causes significant rearrangements in gene expression both at the transcriptional and translational levels. Since eL38, as a component of the ribosome, is involved in the operation of the cell’s translational machinery, the activation of a large number of genes associated with the regulation of translation at both of these levels is quite expected. The increased TEs of these genes should help the cell to adapt its proteome to new conditions, determined by a reduced level of eL38 and associated abnormalities. Regarding the finding on decreased TEs of genes involved in the activation of Hox genes during differentiation, gained with eL38 knocked down HEK293 cells, it is consistent with previously reported data showing that in somites of mutant mouse embryos with eL38 haploinsufficiency, the content of specific subsets of Hox gene mRNAs in polysomes is markedly reduced [9]. It is known that Hox genes are the main regulators of morphology of the axial skeleton [14,15], which are activated in differentiating cells during development [16]. We did not find any statistically significant differences in the TEs of Hox genes between eL38-deficient and control cells, most likely due to the very low content of their mRNAs in HEK293 cells, which is natural, because they do not differentiate. Nevertheless, we can argue that a decrease in TEs of a number of genes involved in the activation of Hox genes should lead to a reduction of the total expression of Hox genes. Apparently, during differentiation in eL38-deficient cells, there is a complex pathway of suppressing the expression of subsets of Hox genes, which is implemented by decreasing the level of proteins involved in their activation. In addition, the reduced content of eL38 mRNA may result in a deficiency of regulatory peptides encoded by its upstream open reading frames, whose sequences can be gained from Ribo-seq data collected in the Trips-Visual Transcriptome browser [17], and thus lead to the impaired expression of genes controlled by them, among which there may be those implicated in the activation of Hox genes. Accordingly, the consequences of decreased translational activities of Hox genes could probably be the formation of a shortened tail and the presence of additional pairs of chest ribs in eL38-deficient mice [9]. However, not all skeletal anomalies observed in these animals can be attributed to impaired Hox gene expression. For example, excessive ossification is most likely related to the enhanced transcription of the *BMP2* and *BMP6* genes revealed in [11], which are involved in skeletal regeneration and the morphogenesis of cartilage and bones. In addition, decreased translational efficiencies of the collagen genes *COL2A1* and *COL6A1* can also contribute to the disruption of the process that regulates bone formation during development [18,19,20]. Possibly, increased transcriptional activities of the *BMP2* and *BMP6* genes and reduced translational efficiencies of the *COL2A1* and *COL6A1* genes are part of a cascade of regulatory events triggered by a decrease in the level of eL38, which ultimately leads to those abnormalities in the axial skeleton that have been observed in mutant mice [8,9].

Our finding that eL38-lacking ribosomes present in eL38 knocked down cells are active in translation, indicates that eL38, unlike many other ribosomal proteins [21], is not critical for the assembly and export of 60S subunits to the cytoplasm and their subsequent participation in translation in the composition of ribosomes, which is consistent with earlier observations [9,11]. Along with this, a decrease in the eL38 level somehow affects the productivity of translation in cells, as evidenced by the increase in the expression levels at this stage of many genes directly related to this process, observed in our study. It was unlikely that this increase was caused by the impaired regulatory effect of eL38, free or in 60S ribosomal subunits, on translation of the respective mRNAs due to a deficiency of the protein itself or 60S subunits with a complete set of proteins. Indeed, ribosomal proteins are hardly to have such an effect, because they are concentrated mainly in the nucleus and nucleoli, and their levels in the cytoplasm, where they are bound to karyopherins [22], are much lower. As for eL38 in the 60S ribosomal subunit, the latter does not interact with mRNAs and begins to participate in translation only from the moment of its attachment to the 48S pre-initiation complex, which results in the formation of the 80S initiation complex. Thus, all the above makes it extremely improbable that eL38, being in a free state or in the 60S subunit, is directly involved in the regulation of translation of the certain mRNAs.

On the other hand, our observation that genes whose mRNAs have CDSs much longer than the average length of mRNA CDSs of protein-coding genes in the human genome are predominant among the down-regulated GDTEs, suggests that ribosomes without eL38 are less active in translation than those containing the entire set of proteins. The slight but still distinct difference in the polysome profiles between HEK293 cells knocked down of eL38 and those treated with non-targeting siRNA also indicates the effect of the protein deficiency on global translation. This may result from the less efficient formation of 80S initiation complexes with the involvement of 60S subunits lacking eL38, as compared to the way it occurs with the participation of normal 60S subunits, due to the possibly lower affinity of the former for eIF6, the translation initiation factor that ensures the 60S subunit joining to the 48S preinitiation complex [23,24]. The eIF6 binding site is located on the 60S subunit near eL38, and the lack of the protein in the 60S subunit may affect its affinity for the factor. Alternatively, the absence of eL38 may allosterically change the structure of the 60S subunit tunnel, making ribosomes without eL38 less efficient in translating certain mRNA regions due to the slowing down of the passage of respective nascent peptides through the tunnel. This may lead to the stalling of ribosomes in these regions (see, for example, [25]), followed by the collisions of ribosomes with and without eL38 and the interruption of translation. Obviously, such disorders in the synthesis of polypeptide chains of proteins should cause a deficiency of gene products, whose mRNAs have long CDSs, which will be felt by cells. Therefore, we speculate that under these conditions cells turn on internal compensatory mechanisms and activate genes responsible for main cellular processes, including the genes that encode RNA processing and translation factors so as to diminish the consequences of imbalance of proteins encoded by genes that appear down-regulated. The activation of these genes at the translational level may occur by some common, as yet unknown mechanism based on the interaction of the 5′-UTRs of their mRNAs with specific, as yet undiscovered signaling proteins or peptides, which should be switched on at a reduced level of eL38 in cells. Our hypothesis on the existence of such a signaling mechanism is in line with the concept of selective translation by specialized ribosomes, in particular, ribosomes heterogeneous in the eL38 protein.

Thus, the application of Ribo-seq to eL38 knocked down HEK293 cells made it possible to determine genes whose expression at the translational level was sensitive to the content of eL38 in ribosomes. Findings from the identification of genes with altered translational efficiencies and from analysis of the pathways, in which these genes are involved, shed light on the possible causes of some abnormalities in mammals deficient in this ribosomal protein. Determining the mechanism enhancing the translation of certain mRNAs, along with the one that suppresses the translation of other mRNAs, triggered by cells with the appearance of 60S ribosomal subunits without eL38, and identifying of the key players providing the respective processes could become the next frontier for further research.

## 4. Materials and Methods

### 4.1. Preparation of siRNAs, Cell Culturing, eL38 mRNA Knockdown and Determination of eL38 and Its mRNA Levels

Oligoribonucleotides used as siRNAs were the same as given in [11]. HEK293 cells (ATCC CRL-1573) were cultured, transfected with the siRNAs and harvested as described in [26]. Cell lysis, the centrifugation of the lysates in a sucrose density gradient, and an analysis of the polysome profiles were performed in accordance with [27]. The contents of ribosomal protein eL38 and reference proteins GAPDH and eS4 in transfected cells and in sucrose gradient fractions were measured by western blotting using rabbit eL38-specific antibodies (#PA5-88313) (Thermo Fisher Scientific, Waltham, MA, USA), rabbit GAPDH-specific antibodies (#60004-1-Ig) (Proteintech, Rosemont, IL, USA) and home-made rabbit antiserum against human ribosomal protein eS4, as described in [11,26].

### 4.2. Ribo-seq Procedures

Ribo-seq was carried out as described [12] with minor modifications. In a typical experiment performed in three biological replicates, HEK293 cells grown in 6-cm Petri dishes were transfected with specific siRNAs against the eL38 mRNA (3 dishes) and with non-targeting siRNA (3 dishes). Two days after the transfection, harringtonine (Invitrogen, Waltham, MA, USA) and cycloheximide (Fluka, Buchs, Switzerland) were consecutively added to the cellular medium to final concentrations of 2 µg/mL and 100 µg/mL, respectively, after which the cells were harvested on ice and pelleted by centrifugation (500× *g*, 1 min, 4 °C). One-third of the cell pellet was resuspended in TRIzol (Invitrogen) and used for total RNA extraction according to the manufacturer’s protocol, followed by the utilization of the obtained sample for RNA-seq; the remaining cell pellet was used for subsequent Ribo-seq procedures. Cells were lysed in 300 µL of buffer 20 mM Tris-HCl (pH 7.5) containing 150 mM NaCl, 5 mM MgCl_2_, 1 mM DTT, 100 µg/mL of cycloheximide, 1% Triton-X100 and 25 U/mL of Turbo DNaseI (Ambion, Waltham, MA, USA). The lysate was then consecutively treated with RNase I and SUPURase-In RNase Inhibitor (both from Invitrogen) according to [12] and centrifuged through a sucrose cushion in a half-cut polycarbonate ultracentrifuge tube in a SW 60 Ti rotor (Beckman Coulter, Brea, CA, USA) at 200,000× *g* for 4 h at 4 °C to pull down the monoribosomes. RNA fragments were recovered from the pellet using the miRNeasy kit (Qiagen, Hilden, Germany) and subjected to the size selection procedure by separating them by 10% denaturing polyacrylamide gel electrophoresis and collecting the fragments in the range of 26–34 nt in length. The resulting RNA fragments were 5′-end phosphorylated and 3′-end dephosphorylated utilizing T4 polynucleotide kinase (NEB) according to the respective manufacturer’s instructions. DNA-libraries were prepared from the obtained RNA fragment samples using the 5500 SOLiD Fragment Library Core Kit (Life Technologies, Waltham, MA, USA) (replicate 1) and the NEBNext Ultra DNA Library Prep Kit (New England Biolabs, Ipswich, MA, USA) (replicates 2 and 3) according to the manufacturer’s instructions. Next generation sequencing (NGS) was carried out in SB RAS Genomics Core Facility (ICBFM SB RAS, Novosibirsk). Replicate 1 was sequenced on the SOLiD 5500xl platform (Life Technologies) and replicates 2 and 3, were sequenced on the HiSeq2500 (Illumina, San Diego, CA, USA). The Ribo-seq read data reported in this study were submitted to the GenBank under the BioProject accession PRJNA657546 (SRR13993919–SRR13993924); the RNA-seq read data have been submitted to GeneBank earlier under the BioProject accessions PRJNA657546 (SRR12494554–SRR12494556,) and PRJNA611889 (SRR11285589, SRR11285590 and SRR11285592) [11].

### 4.3. NGS Data Analysis

Fastq reads were analyzed using tools of the CLC GW 12.0 software (Qiagen). Reads were filtered for both the quality (by default) and adapter sequences, and then mapped to the human reference genome (hg38) with Ensembl annotation GRCh38.93 by the RNA-seq analysis tool (Strand specific = Forward and other parameters by default). The resulted BAM files were sorted, indexed and used for subsequent analysis steps. RNA-seq analysis carried out using the DESeq2 and other Bioconductor packages has been described in [11]. The quality-check of Ribo-seq data was performed with the ribosomeProfilingQC (1.2.0,) and RiboProfiling (1.20.0) packages. The analysis of the TEs of genes was carried out by the DESeq2 using likelihood ratio test (LRT) as described in the systemPipeR package vignette [28]. To this end, a designed formula (LRT = ~batch + assay + condition + assay:condition) was used, where batch one corresponds to replicate 1 and batch two to replicates 2 and 3, assays are either Ribo-seq or RNA-seq, and conditions designated as either “N” or “KD” correspond to HEK293 cells treated with either non-targeting or eL38 mRNA-specific siRNAs, respectively. The batch parameter takes into account various technical errors, including those caused by using data from different sequencing platforms, when determining the TE values of genes. In the above formula, the assay:condition reflects the ratio of ratios corresponding to (Ribo_A1/mRNA_A1)/(Ribo_M1/mRNA_M1) as described in [28], where the Ribo_A1 and Ribo_M1 parameters are Ribo-seq data for the conditions “KD” and “N”, respectively, and the mRNA_A1 and RNA_M1 parameters are RNA-seq data for the same condition types. The resulting gene set with DESeq2-estimated TEs was restricted to a subset with absolute values of LFCs > 0.585, *p*.adj < 0.1 and baseMean > 100. Genes satisfying the above limitations were assigned to GDTEs and used for further analysis.

### 4.4. Determining the GDTEs-Associated Processes

The GO enrichment analysis of GDTEs against the GO terms of the biological processes, cellular components and molecular functions categories was performed using the online-based resource www.geneontology.org (accessed on 15 March 2021) [29] with the application of the Fisher’s Exact test type and the False Discovery Rate (FDR) correction. Only the GO terms with the values of Fold enrichment > 3 and FDR < 0.01 were taken into consideration. Pathway analysis was performed using the ReactomePA package (1.34.0) [30] with default parameters. The *p*.value.cutoff parameter was assigned as 0.05. Other manipulations were performed with custom R scripts. The visualization of plots was predominantly performed using the ggplot2 package.

## Figures and Tables

**Table 1:** Genes that were activated at the translational level in eL38-deficient HEK293 cells.

#	Gene Symbol	Description	*p*.adj	Shrunken LFC
1	*ATP5MG*	ATP synthase membrane subunit g	0.012694951	4.312883344
2	*TPM4*	tropomyosin 4	0.012694951	2.407078538
3	*TUBA1A*	tubulin alpha 1a	0.028524939	2.303007559
4	*NME1*	NME/NM23 nucleoside diphosphate kinase 1	0.010574469	2.189657665
5	*CADM1*	cell adhesion molecule 1	0.007530612	2.027829748
6	*LAMP2*	lysosomal associated membrane protein 2	0.00925901	1.908361642
7	*CDK1*	cyclin dependent kinase 1	0.01847032	1.803062543
8	*RCC1*	regulator of chromosome condensation 1	0.007265371	1.727042562
9	*RPL26*	ribosomal protein L26	0.011706077	1.636955215
10	*RPL10*	ribosomal protein L10	0.000538004	1.635221166
11	*NUP43*	nucleoporin 43	0.008195855	1.632862465
12	*FUBP3*	far upstream element binding protein 3	0.029189622	1.617421008
13	*FBXW11*	F-box and WD repeat domain containing 11	0.076761322	1.57234785
14	*PRPS2*	phosphoribosyl pyrophosphate synthetase 2	0.029868456	1.570681683
15	*RARS*	arginyl-tRNA synthetase	0.007265371	1.510380471
16	*HNRNPA3*	heterogeneous nuclear ribonucleoprotein A3	0.009556945	1.429504559
17	*SLC25A5*	solute carrier family 25 member 5	0.000250642	1.426609786
18	*EIF4A2*	eukaryotic translation initiation factor 4A2	0.032395672	1.387976198
19	*NCBP2*	nuclear cap binding protein subunit 2	0.054499316	1.366077171
20	*BCCIP*	BRCA2 and CDKN1A interacting protein	0.040935557	1.33229145
21	*FAU*	FAU, ubiquitin like and ribosomal protein S30 fusion	0.074353133	1.292640825
22	*UQCRB*	ubiquinol-cytochrome c reductase binding protein	0.044358822	1.283025687
23	*PMPCB*	peptidase, mitochondrial processing beta subunit	0.010250639	1.27205479
24	*PDHA1*	pyruvate dehydrogenase E1 alpha 1 subunit	0.00218117	1.269166906
25	*MAPRE1*	microtubule associated protein RP/EB family member 1	0.049156453	1.266782661
26	*SKP1*	S-phase kinase associated protein 1	0.071604825	1.254078271
27	*HMGB1*	high mobility group box 1	0.012694951	1.230704281
28	*ACAT1*	acetyl-CoA acetyltransferase 1	0.084656497	1.229396761
29	*SHMT2*	serine hydroxymethyltransferase 2	0.029915865	1.229351493
30	*COPZ1*	coatomer protein complex subunit zeta 1	0.074353133	1.215072132
31	*USP14*	ubiquitin specific peptidase 14	0.010498702	1.178426319
32	*RPS15A*	ribosomal protein S15a	0.069434337	1.171489242
33	*SDHC*	succinate dehydrogenase complex subunit	0.076718561	1.153724537
34	*GLYR1*	glyoxylate reductase 1 homolog	0.075169122	1.148655113
35	*OAT*	ornithine aminotransferase	0.005882567	1.134150228
36	*VPS29*	VPS29, retromer complex component	0.088864364	1.122312686
37	*DNAJC21*	DnaJ heat shock protein family (Hsp40) member C21	0.096374154	1.120757635
38	*B2M*	beta-2-microglobulin	0.083259285	1.108883017
39	*FOXC1*	forkhead box C1	0.06145435	1.108340457
40	*SKI*	SKI proto-oncogene	0.088864364	1.102900252
41	*TGOLN2*	trans-golgi network protein 2	0.037609589	1.101849221
42	*FBL*	fibrillarin	0.044358822	1.097579167
43	*TFAP2A*	transcription factor AP-2 alpha	0.049183194	1.09566041
44	*PRKAR1A*	protein kinase cAMP-dependent type I regulatory subunit alpha	0.055044332	1.09362372
45	*NEFL*	neurofilament light	0.063550637	1.075315115
46	*PTTG1*	pituitary tumor-transforming 1	0.03996427	1.073127863
47	*PREP*	prolyl endopeptidase	0.099910871	1.06731661
48	*EIF3L*	eukaryotic translation initiation factor 3 subunit L	0.065101199	1.062215988
49	*HSPA9*	heat shock protein family A (Hsp70) member 9	0.013159468	1.049985495
50	*SRSF1*	serine and arginine rich splicing factor 1	0.081526317	1.034177714
51	*CDC23*	cell division cycle 23	0.035728724	1.033849332
52	*NAE1*	NEDD8 activating enzyme E1 subunit 1	0.054499316	1.0329236
53	*CDC37*	cell division cycle 37	0.076761322	1.005715312
54	*NCAPG2*	non-SMC condensin II complex subunit G2	0.048942052	1.000694023
55	*MTREX*	Mtr4 exosome RNA helicase	0.054604073	0.99292805
56	*CCT7*	chaperonin containing TCP1 subunit 7	0.002282515	0.977419566
57	*KAT7*	lysine acetyltransferase 7	0.045918547	0.974711617
58	*RPS24*	ribosomal protein S24	0.007265371	0.970173253
59	*HES1*	hes family bHLH transcription factor 1	0.068638154	0.959729472
60	*RBM17*	RNA binding motif protein 17	0.076761322	0.946115122
61	*RPS3A*	ribosomal protein S3A	0.098865311	0.921705513
62	*AUP1*	AUP1. lipid droplet regulating VLDL assembly factor	0.067871964	0.904287613
63	*HNRNPA1*	heterogeneous nuclear ribonucleoprotein A1	0.000431183	0.895925461
64	*RPL5*	ribosomal protein L5	0.001910138	0.895501706
65	*SUCLG1*	succinate-CoA ligase alpha subunit	0.065734544	0.868052522
66	*BUB1*	BUB1 mitotic checkpoint serine/threonine kinase	0.056010491	0.849315094
67	*INTS14*	integrator complex subunit 14	0.069509177	0.843657405
68	*CCT4*	chaperonin containing TCP1 subunit	0.002282515	0.835602103
69	*INTS13*	integrator complex subunit 13	0.099231448	0.828717986
70	*PLS3*	plastin 3	0.040594481	0.8130572
71	*EIF5*	eukaryotic translation initiation factor 5	0.03403791	0.812449142
72	*NARS*	asparaginyl-tRNA synthetase	0.037609589	0.809858958
73	*CCT3*	chaperonin containing TCP1 subunit 3	0.057566318	0.806374214
74	*KIF22*	kinesin family member 22	0.076761322	0.792676124
75	*CCT6A*	chaperonin containing TCP1 subunit 6A	0.028782703	0.786750634
76	*SEPTIN7*	septin 7	0.062370622	0.77110045
77	*PAICS*	phosphoribosylaminoimidazole carboxylase and phosphoribosylaminoimidazolesuccinocarboxamide synthase	0.09282529	0.762744164
78	*ACBD3*	acyl-CoA binding domain containing 3	0.096199008	0.738281486
79	*EIF4H*	eukaryotic translation initiation factor 4H	0.05417421	0.718503713
80	*EIF2S3*	eukaryotic translation initiation factor 2 subunit gamma	0.052916229	0.704489885
81	*FKBP4*	FK506 binding protein 4	0.048451346	0.697700308
82	*EEF1A1*	eukaryotic translation elongation factor 1 alpha 1	0.001499086	0.663548054
83	*XRCC6*	X-ray repair cross complementing 6	0.017242388	0.644500151
84	*HNRNPA2B1*	heterogeneous nuclear ribonucleoprotein A2/B1	0.052916229	0.611694961

**Table 2:** Genes that were suppressed at the translational level in eL38-deficient HEK293 cells.

#	Gene Symbol	Description	*p*.adj	Shrunken LFC
1	*ATN1*	atrophin 1	0.000250642	−2,383,968,333
2	*HHIPL1*	HHIP like 1	0.099910871	−2,108,283,211
3	*LAMA5*	laminin subunit alpha 5	1.04737E-07	−2,099,435,635
4	*NOMO3*	NODAL modulator 3	0.034998577	−1,772,216,098
5	*GSK3A*	glycogen synthase kinase 3 alpha	0.086893975	−1,657,765,364
6	*OCIAD2*	OCIA domain containing 2	0.041499491	−1,602,956,347
7	*AUTS2*	AUTS2, activator of transcription and developmental regulator	0.007265371	−1,450,228,921
8	*CAMSAP1*	calmodulin regulated spectrin associated protein 1	0.020568057	−1,399,881,818
9	*PRELID1*	PRELI domain containing 1	0.022809358	−1,365,693,298
10	*ZNF274*	zinc finger protein 274	0.01422397	−1,355,991,291
11	*GAK*	cyclin G associated kinase	0.010498702	−1,351,541,744
12	*LTBP1*	latent transforming growth factor beta binding protein 1	0.000250642	−1,336,690,238
13	*MAX*	MYC associated factor X	0.040594481	−1,320,705,011
14	*HIST1H2BC*	histone cluster 1 H2B family member c	0.064581433	−1,314,815,037
15	*EDC4*	enhancer of mRNA decapping 4	0.042249906	−1,307,590,139
16	*ASCC2*	activating signal cointegrator 1 complex subunit 2	0.029868456	−1,256,837,357
17	*ENOSF1*	enolase superfamily member 1	0.081515108	−1,250,428,661
18	*CTCF*	CCCTC-binding factor	0.007366565	−1,239,742,061
19	*IQGAP3*	IQ motif containing GTPase activating protein 3	0.033902778	−119,141,635
20	*RPLP1*	ribosomal protein lateral stalk subunit P1	0.002534611	−1,125,473,816
21	*C6orf106*	chromosome 6 open reading frame 106	0.052916229	−1,106,301,158
22	*AEN*	apoptosis enhancing nuclease	0.010498702	−1,105,573,856
23	*CDK5RAP2*	CDK5 regulatory subunit associated protein 2	0.055629954	−1,096,112,547
24	*ATP13A2*	ATPase cation transporting 13A2	0.015260908	−1,083,453,368
25	*ITPR3*	inositol 1,4,5-trisphosphate receptor type 3	0.052068978	−1,046,481,817
26	*COL6A1*	collagen type VI alpha 1 chain	0.059954251	−1,046,201,279
27	*CYB5B*	cytochrome b5 type B	0.061939362	−1,045,026,726
28	*FASN*	fatty acid synthase	0.020429889	−1,006,556,157
29	*TBRG4*	transforming growth factor beta regulator 4	0.031530019	−1,000,246,535
30	*SERP1*	stress associated endoplasmic reticulum protein 1	0.098408591	−0.99348297
31	*SBF1*	SET binding factor 1	0.020568057	−0.991430768
32	*HIST1H4E*	histone cluster 1 H4 family member e	0.029649385	−0.985384473
33	*DDT*	D-dopachrome tautomerase	0.054499316	−0.98064313
34	*FBRS*	fibrosin	0.035039202	−0.969773425
35	*STRN4*	striatin 4	0.089949483	−0.943078161
36	*WDR62*	WD repeat domain 62	0.035792707	−0.923170831
37	*URB2*	URB2 ribosome biogenesis 2 homolog (*S. cerevisiae*)	0.085713486	−0.89953143
38	*PIEZO1*	piezo type mechanosensitive ion channel component 1	0.026333758	−0.891075548
39	*KMT2D*	lysine methyltransferase 2D	0.052916229	−0.889992224
40	*SDCBP*	syndecan binding protein	0.05742347	−0.882057197
41	*SMARCC1*	SWI/SNF related, matrix associated, actin dependent regulator of chromatin subfamily c member 1	0.035728724	−0.870997243
42	*HECTD4*	HECT domain E3 ubiquitin protein ligase 4	0.04412228	−0.868499831
43	*ARHGAP35*	Rho GTPase activating protein 35	0.072048538	−0.86688062
44	*SYMPK*	symplekin	0.019920301	−0.855418984
45	*ABCA3*	ATP binding cassette subfamily A member 3	0.096753082	−0.841808007
46	*HIST1H2AC*	histone cluster 1 H2A family member c	0.038804021	−0.834943336
47	*KIF1B*	kinesin family member 1B	0.057776732	−0.834338228
48	*RETREG3*	reticulophagy regulator family member 3	0.089018972	−0.826365716
49	*CLUH*	clustered mitochondria homolog	0.079547821	−0.822733101
50	*PELP1*	proline, glutamate and leucine rich protein 1	0.072048538	−0.803585995
51	*COL2A1*	collagen type II alpha 1 chain	0.020704772	−0.803529736
52	*CLPTM1*	CLPTM1, transmembrane protein	0.080528426	−0.79290716
53	*NUP210*	nucleoporin 210	0.020704772	−0.78932396
54	*SFSWAP*	splicing factor SWAP	0.094719096	−0.788876906
55	*IGF1R*	insulin like growth factor 1 receptor	0.086783977	−0.784038911
56	*UBE2D2*	ubiquitin conjugating enzyme E2 D2	0.035728724	−0.762644136
57	*MEGF8*	multiple EGF like domains 8	0.062032886	−0.741762602
58	*DDB1*	damage specific DNA binding protein 1	0.09771499	−0.711925724
59	*MDC1*	mediator of DNA damage checkpoint 1	0.042983832	−0.711259755
60	*FLNA*	filamin A	0.007366565	−0.701321881
61	*SUGP2*	SURP and G-patch domain containing 2	0.092506997	−0.67863598
62	*MAGED1*	MAGE family member D1	0.09697418	−0.662214919
63	*UPF1*	UPF1, RNA helicase and ATPase	0.099910871	−0.661569626
64	*CLTC*	clathrin heavy chain	0.061939362	−0.660003656
65	*BAG6*	BCL2 associated athanogene 6	0.074741705	−0.656625382
66	*HMGA1*	high mobility group AT-hook 1	0.076718561	−0.626612021

## Data Availability

The Ribo-seq read data reported in this study were submitted to the GenBank under the BioProject accession PRJNA657546 (SRR13993919–SRR13993924).

## Supplementary Materials

Supplementary Materials can be found at https://www.mdpi.com/article/10.3390/ijms22094531/s1.

## Abbreviations

CDS	coding sequence
GDTE	gene with differential translational efficiency
GO	Gene Ontology
NGS	next generation sequencing
TE	translational efficiency
