# Supplementary material for: Knockdown of the Ribosomal Protein eL38 in HEK293 Cells Changes the Translational Efficiency of Specific Genes
Source: Int J Mol Sci. 2021 Apr 26;22(9):4531. doi: 10.3390/ijms22094531 (PMC8123606; doi:10.3390/ijms22094531)
Supplement: Supplementary file 1 [file ijms-22-04531-s001.zip › ijms-1181981-supplementary.pdf]

Supplementary figures and table

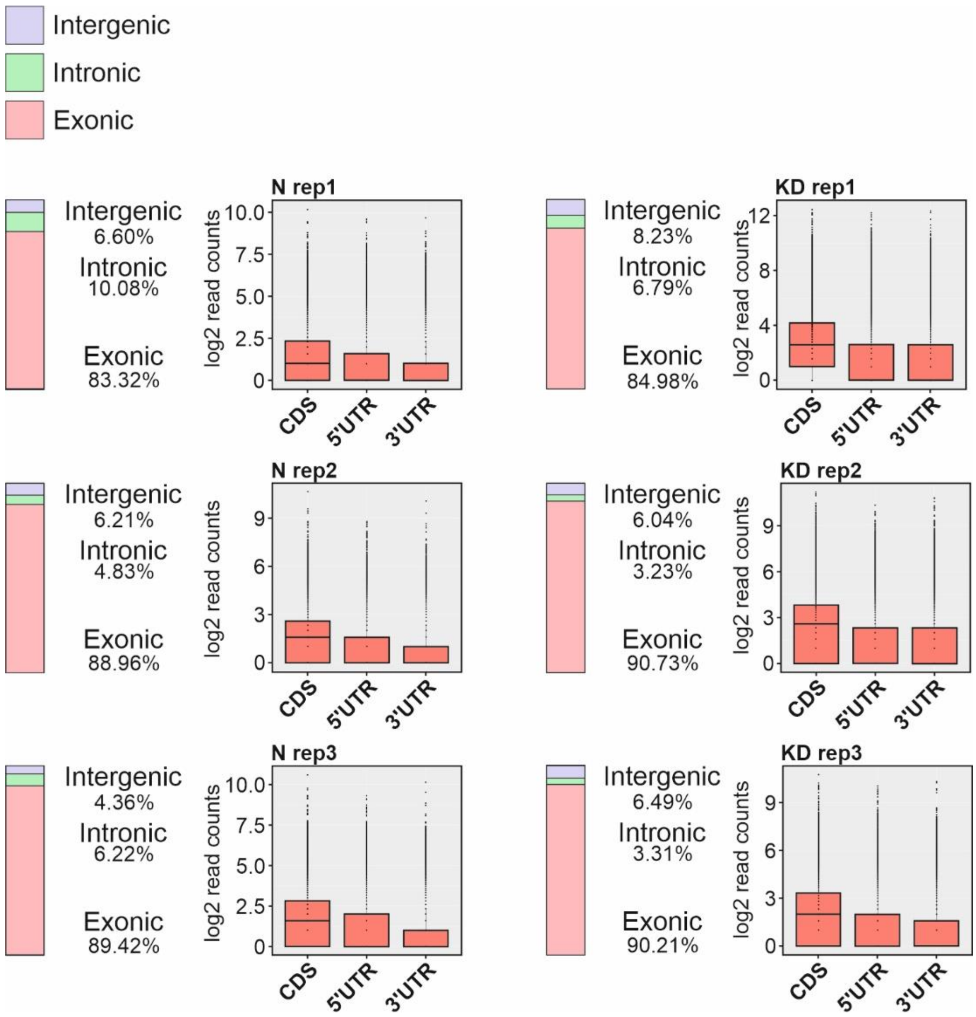

Figure S1. The distribution of Ribo-seq reads across genomic features for each sample generated using Qualimap and RiboProfiling (N and KD correspond to HEK293 cells treated with non-targeting and eL38 mRNA-specific siRNAs, respectively). The bar plots represent the distribution of the reads across intergenic, intronic and exonic regions (see the designations in the upper left corner). The boxplots inside the squares reflect the mean log2 values of the number of reads per a region of protein-coding genes corresponding to the 5'UTR, CDS or 3'UTR of their mRNAs for each sample.

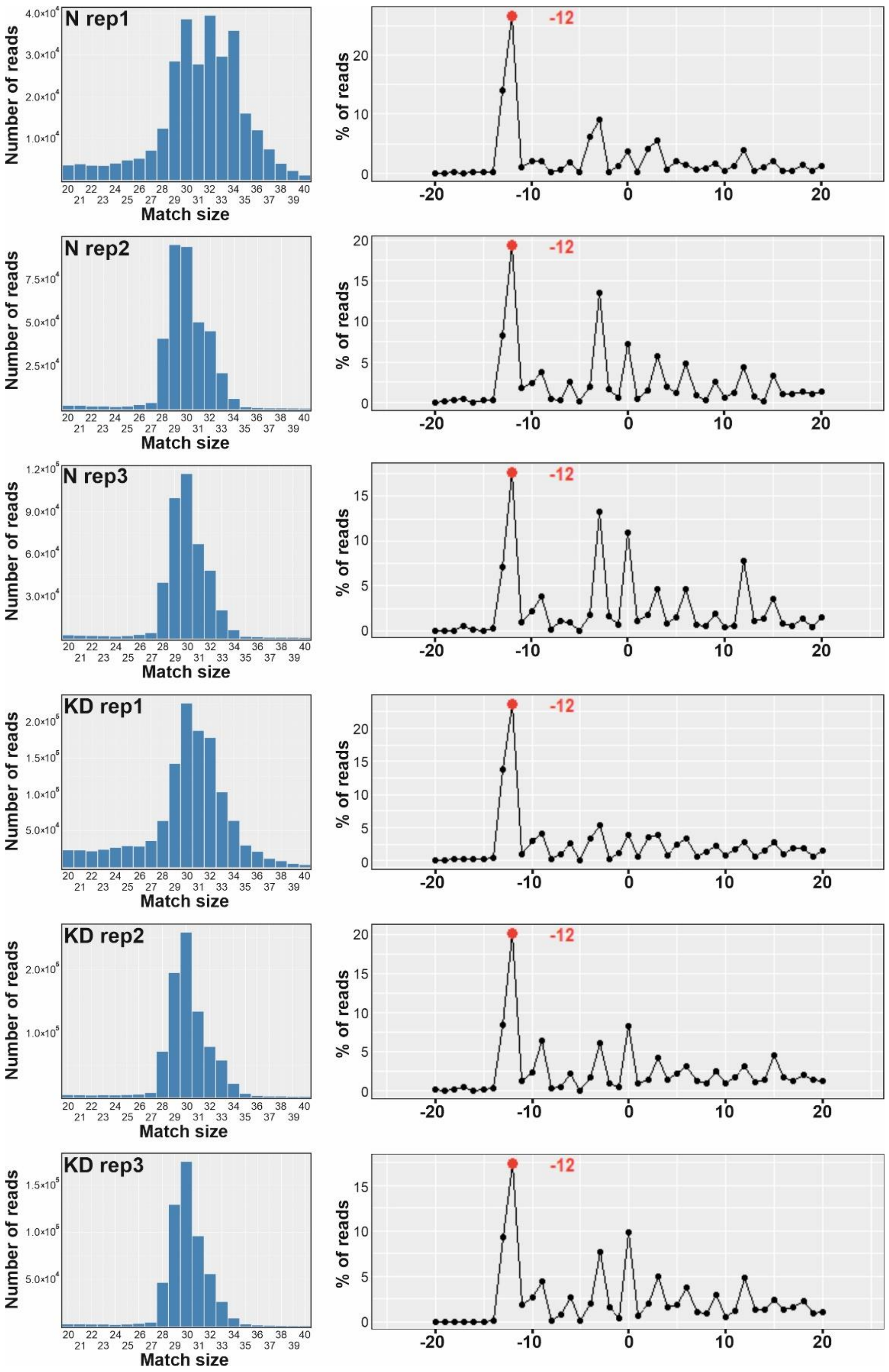

Figure S2. The distribution of Ribo-seq read lengths across all samples and the determination of the P-site offset generated using the RiboProfiling package (N and KD, see the legend to Figure S1). The left panels are histograms of read length distributions obtained using the *histMatchLength* function. The X-axis presents the length of the reads (from 22 to 40 bp), the Y-axis presents the number of Ribo-seq reads. The right parts are the read frequency distributions centered at the translation start site (position 0) of the most abundant protein-coding transcripts. The marked position -12 shows the offset of the position of the 5' read ends from that of the translation start site, and the distance between positions 0 and -12 corresponds to the length of

the regions of mRNAs upstream the start codon, protected by ribosomes from RNase I hydrolysis. The peaks that repeat every three positions are due to the periodicity of the codons in mRNAs.

Table S1. Basic library characteristics (according to FastQC and Qualimap reports).

Sample ID	Internal ID	Batch	Platform	Raw reads	Sequencing mode	Aligned to genes (exonic)
N rep1	5-R07	I	SOLiD 5500 XL		50 SE	105,002
N rep2	8-R01	II	Illumina HiSeq 2500	2 x 14782576	2x100 PE	262,215
N rep3	8-R02	II	Illumina HiSeq 2500	2 x 12996421	2x100 PE	290,964
KD rep1	5-R09	I	SOLiD 5500 XL		50 SE	657,364
KD rep2	8-R05	II	Illumina HiSeq 2500	2 x 13770357	2x100 PE	590,733
KD rep3	8-R06	II	Illumina HiSeq 2500	2 x 10143961	2x100 PE	393,618
